# Revised CT angiography venous score with consideration of infratentorial circulation value for diagnosing brain death

**DOI:** 10.1186/s13613-016-0188-7

**Published:** 2016-09-13

**Authors:** Antoine J. Marchand, Philippe Seguin, Yannick Malledant, Marion Taleb, Hélène Raoult, Jean Yves Gauvrit

**Affiliations:** 1 Department of Radiology and Medical Imaging, University and Regional Hospital Center (CHRU) of Rennes, 2 rue Henri Le Guillou, 35000 Rennes, France; 2 Service d’Anesthésie Réanimation 1, CHU Rennes, 2 rue Henri Le Guillou, 35000 Rennes, France; 3 Université Rennes 1, Rennes, France; 4 Inserm U991, Rennes, France

**Keywords:** Brain death diagnosis, Computed tomography angiography, Confirmatory test, Revised four-point venous score

## Abstract

**Background:**

Computed tomography angiography (CTA) is largely performed in European countries as an ancillary test for diagnosing brain death. However, CTA suffers from a lack of sensitivity, especially in patients who have previously undergone decompressive craniectomy. The aim of this study was to assess the performance of a revised four-point venous CTA score, including non-opacification of the infratentorial venous circulation, for diagnosing brain death.

**Methods:**

A preliminary study of 43 control patients with normal CTAs confirmed that the infratentorial superior petrosal vein (SPV) was consistently visible. Therefore, 76 patients (including ten with decompressive craniectomy) who were investigated with 83 CTAs to confirm clinical brain death were consecutively enrolled between July 2011 and July 2013 at a university centre. The image analysis consisted of recording non-opacification of the cortical segment of the middle cerebral artery and internal cerebral vein (ICV), which were used as the reference CTA score, as well as non-opacification of the SPV. The diagnostic performance of the revised four-point venous CTA score based on the non-opacification of both the ICV and SPV was assessed and compared with that of the reference CTA score.

**Results:**

The revised four-point venous CTA score showed a sensitivity of 95 % for confirming clinical brain death versus a sensitivity of 88 % with the reference CTA score. Non-opacification of the SPV was observed in 95 % of the patients. In the decompressive craniectomy group, the revised four-point CTA score showed a sensitivity of 100 % compared with a sensitivity of 80 % using the reference CTA score.

**Conclusion:**

Compared with the reference CTA score, the revised four-point venous CTA score based on ICV and SPV non-opacification showed superior diagnostic performance for confirming brain death, including for patients with decompressive craniectomy.

## Background

Brain death is defined as the irreversible cessation of all functions of the entire brain [1]. The clinical brain death diagnosis is based on the three brain stem criteria: total and irreversible coma with a known cause, the absence of brain stem reflexes, and apnoea [2]. However, determining these clinical criteria may be hindered by complex or spontaneous motor movements; false-positive triggering of the ventilator [1]; or particular contexts, such as barbituric impregnation, hypothermia, haemodynamic instability, drug intoxication, or immature central nervous system in young children [3]. Thus, a brain death diagnosis may require confirmatory tests, which are mandatory in several countries. Ideally, these tests would explore the entire brain functions (and confirm whole brain death) including supra-tentorial cortex (neocortical brain death) and brain stem (brain stem death). Nevertheless, most of them explore only cortex, notably computed tomography angiography (CTA) when used as actually preconized. This is a critical issue particularly when clinical diagnosis is uncertain [4, 5]. There are two types of ancillary tests. The first type analyses brain functions and includes EEG, auditory-evoked potentials, and somatosensory-evoked potentials. However, these tests present the same limitations as clinical examinations in cases of drug impregnation or hypothermia [6]. Furthermore, the tools required to perform and analyse cerebral functions are limited to the largest centres [7].

The second type of ancillary test aims to show cerebral circulation arrest and includes digital subtraction angiography (DSA), computed tomography angiography (CTA) [8], transcranial Doppler [9], encephalic scintigraphy, and MR angiography [10–12].

In European countries, CTA is performed in approximately 40 % of cases that require an ancillary test because of either a legal requirement or the failure to perform a clinical diagnosis [13, 14]. Computed tomography has the advantages of being widely available and less invasive than the reference digital subtraction angiography (DSA), less time-consuming than MR angiography or encephalic scintigraphy, and less dependent on the operator than transcranial Doppler. Finally, CTA is the only test that allows for simultaneous pre-harvesting body scanning [15]. Few CTA scores have been proposed for diagnosing brain death [8, 15], and the most recent and currently used consists of four criteria: the non-opacification of the cortical segment of each middle cerebral artery (C-MCA) and the non-opacification of each internal cortical vein (ICV), with the latter being the most sensitive [15]. However, this score suffers from an imperfect and variable sensitivity of 73–96 % [15–18] and is limited in the context of decompressive craniectomy due to an intracranial hypertension decrease [19]. Moreover, the reference CTA score only includes an analysis of the supratentorial circulation, although a clinical brain death diagnosis relies on infratentorial-related function arrest, which is assessed in clinical practice by the absence of brain stem reflexes.

As a consequence, we elaborated a new score to assess whole brain circulation stop and to increase sensitivity for promoting CTA as the preferred ancillary test in diagnosing brain death.

Thus, the aim of this study was to assess the diagnostic performance of a revised four-point brain death CTA score that relies on the non-opacification of both the ICV (ever used in the precedent score) and infratentorial superior petrosal vein (SPV) (new criterion) compared with the previous reference CTA score.

## Methods

### Patient selection

Institutional review board approval was obtained prior to the study (committee’s reference number 13.61). Between July 1, 2011, and July 1, 2013, in a university centre, 76 consecutive patients were examined using CTA to confirm cerebral circulation arrest after a clinical diagnosis of brain death. The clinical diagnosis of brain death was made by trained intensivists according to international guidelines [20]. As recommended, CTA was performed at least 6 h after clinical diagnosis and was repeated 6 h later if the reference CTA score did not confirm cerebral circulation arrest [21].

### Preliminary anatomical study of infratentorial veins to develop a revised four-point venous CTA score

**Fig. 1:**
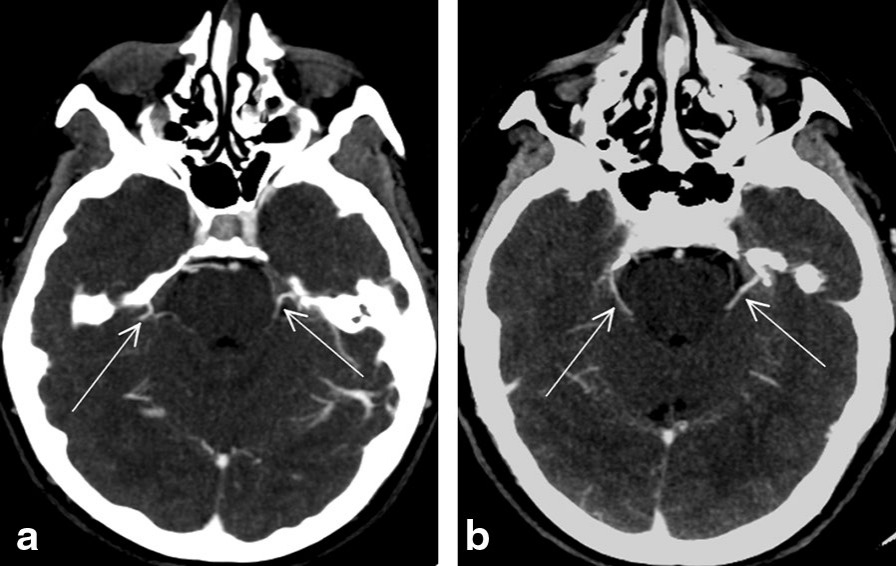
CTA axial views (**a**, **b**) of a control patient. *Arrows* indicate SPV

A preliminary anatomical study aimed to assess the anatomical features of venous circulation, particularly infratentorial features, for selecting the most constantly visible veins to develop a new and reliable venous CTA score. For this, 43 control patients (11 men and 32 women, mean age 38.6, range 17–66 years) were examined using CTA based on clinical suspicion of thrombophlebitis, not confirmed by a CTA that was normal. The analysis classified the ICV and SPV (Fig. 1) as exhibiting complete visibility, visibility with hypoplasia, or lack of visibility [22]. The SPV is described in anatomical studies as one of the largest infratentorial venous stems [22, 23] and is of particular interest because it receives the largest number of tributary veins from the brain stem: the pontotrigeminal, transverse pontine, cerebellopontine fissure, and middle cerebellar peduncle veins. The last portion of the SPV, formed by a single vein segment or by the union of several veins, drains into the superior petrosal sinus. The basilar artery was not assessed because of a previously demonstrated low sensitivity of approximately 80–85 % [15, 16].

The ICV was easily observed and was normotrophic in all the patients, confirming previous studies [8]. The SPV, vessel that was not usually described by radiologists, was observed in all the patients, although it was found to be hypotrophic in 26 % of cases. Moreover, our findings confirmed published reports of varying SPV positions between the lateral or medial sides of the internal acoustic meatus [22–24]. Finally, the SPV and ICV were consistently visible in the control patients.

### Collection of clinical and investigative data related to brain death patients

Data on the following variables were collected: age, sex, origin of brain death, and history of decompressive craniectomy. The results of the apnoea test were provided. Physiological and biological parameters that may interfere with a diagnosis of brain death and the results of the CTAs were recorded at the time that CTA was performed. Finally, catecholamine use and dosage were reported.

### CTA imaging

CTAs were performed using the Lightspeed multidetector GE scanner with 64 rows (GE Healthcare, Waukesha, WI). We followed the recommendations of the French Society of Neuroradiology [21] for the CTA protocol, with an initial spiral acquisition without contrast medium, a second scanning spiral 20 s after the injection of contrast medium to assess intravenous injection, and a final phase 60 s after the injection to study the opacification of the cerebral vessels.

The window centre was preset at 40 Hounsfield units (HU) with a width of 100 HU.

The same CTA protocol was used for the control patient group and the clinical brain death patients, with a spiral acquisition starting 60 s after intravenous medium contrast injection.

### Image analysis

For both the patients with clinical brain death and the control patients, a consensus review of the CTA images was conducted by two trained radiologists with 10 years of experience. The visibility of the ICV, C-MCA, and infratentorial SPV was classified as opacification or non-opacification.

### Statistical analysis

The revised four-point venous score was based on the non-opacification of both the ICV and SPV, with a positive score indicating complete non-opacification. Using the previous reference four-point CTA score [15], a positive score indicated that both the ICV and C-MCA were non-opacified. The scores for each patient were determined by both examining radiologists. The sensitivity of each score to determine cerebral circulation arrest was calculated using a clinical brain death diagnosis as the benchmark for analysis.

## Results

### Patient characteristics

The study included 76 patients, of whom 47 were male (62 %), with a mean age of 52 ± 16 years and a mean initial Glasgow coma score of 7 ± 4. The origins of brain death were intracranial aneurysm (*n* = 25), traumatic brain injury (*n* = 24), stroke (*n* = 10), cerebral haemorrhage (*n* = 9), cerebral anoxia (*n* = 7) and infection (*n* = 1). The results of the apnoea test showed a mean arterial PCO_2_ equal to 80 ± 19 mmHg; however, data were not reported in 26 patients because of oxygen desaturation (*n* = 20), haemodynamic instability (*n* = 3), pregnancy (*n* = 1), or missing data (*n* = 2). Eight of the 76 included patients had decompressive craniectomy.

Physiological parameters recorded at the moment of brain death are provided in Table 1.

**Table 1:** Physiological parameters and catecholamine used at the time of CT angiography (*n* = 76)

Body core temperature (°C)	36.8 ± 1.1 [32.8–39.9]^a^
Mean arterial pressure (mmHg)	80 ± 19
Natraemia (mmol/l)	147 ± 7
PaCO_2_ (mmHg)	37 ± 10
Haemoglobin (g/dl)	10.7 ± 2.3
PaO_2_/FiO_2_	326 ± 105
Diabetes insipidus	59 (78 %)
Catecholamine use^b^	72 (86 %)
Catecholamine dose (µg/kg min^−1^)	0.04 ± 0.06

CTA, computed tomography angiography; PaCO_2_, carbon dioxide arterial pressure; PaO_2_, oxygen arterial pressure; FiO_2_, inspiratory oxygen fraction

^a^Mean ± SD [lower bound − upper bound]

^b^Norepinephrine, *n* = 67; epinephrine, *n* = 1; norepinephrine–dobutamine, *n* = 3; norepinephrine–epinephrine, *n* = 1

**Fig. 2:**
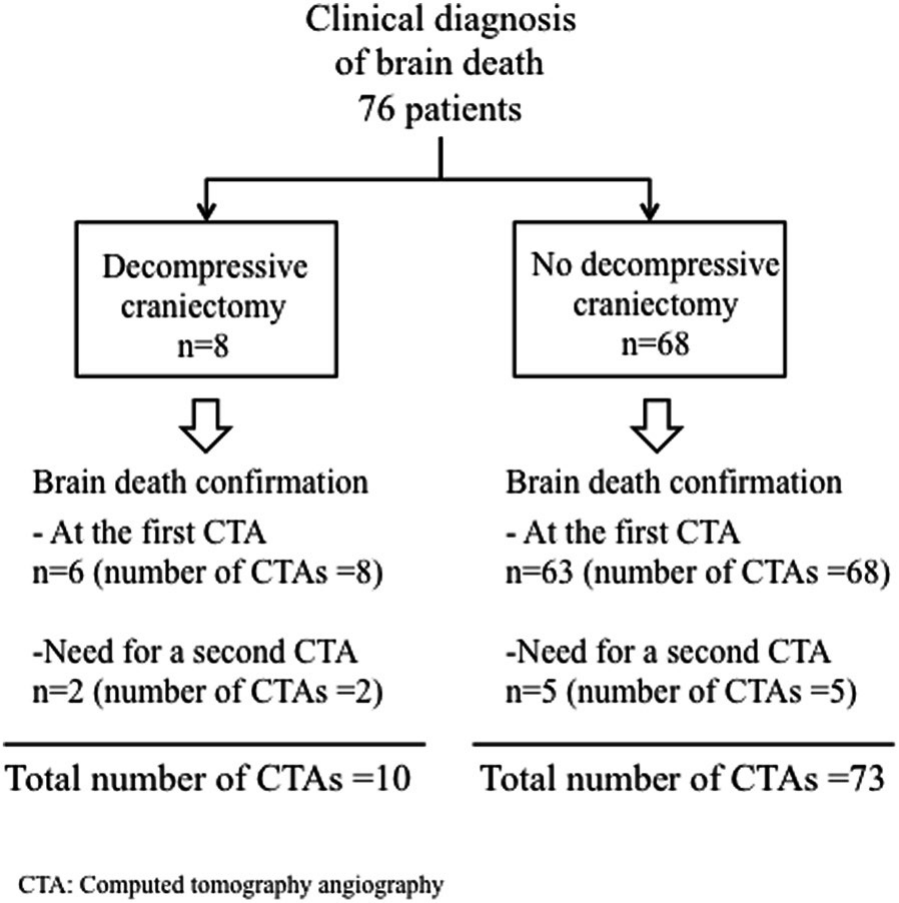
Study’s flowchart

A second CTA was performed six hours after the first for seven of the 76 included patients because the first CTA did not confirm brain death according to the reference four-point CTA score. Finally, 83 CTAs were analysed including a total of ten CTAs in patients with decompressive craniectomy (Fig. 2).

### Diagnostic performance of brain death CTA scores

The second scanning spiral realized 20 s after the injection of contrast medium assessed effective intravenous injection in all the cases.

The reference CTA score and the revised four-point venous CTA score confirmed cerebral circulation arrest (positive score) with sensitivities of 88 % (73/83) and 95 % (79/83), respectively.

**Fig. 3:**
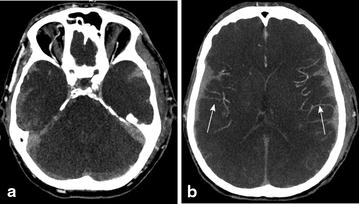
CTA axial views of a clinically brain-dead patient with a lack of SPV opacification (**a**) despite an enhanced bilateral C-MCA (**b**) (*arrows*)

Six CTAs examinations showed persistent opacification of the C-MCA (with negative reference CTA score) associated with non-opacification of the SPV (Fig. 3). Different aetiologies were described in these six cases: two CTAs were performed in patients with infratentorial haemorrhage, one in cerebral anoxia, one in supratentorial ballistic injury, and two in patients with decompressive craniectomy. These six cases of false-negative reference CTA scores are described for each vessel in Table 2.

**Table 2:** CTAs with persistent opacified vessels among patients with a clinical brain death diagnosis (*n* = 83)

Vessel type	CTA examination(s) in which vessel type is still opacified
2 C-MCA (left and right)	8
1 C-MCA (left or right)	10
ICV	3
BA	15
SPV	4

Data are expressed as *n*. Considering ICV and SPV, there was no case where only one vessel side (left or right) was enhanced or not contrary to C-MCA

*C-MCA* cortical segment of middle cerebral artery, *ICV* internal cerebral vein, *BA* basilar artery, *SPV* superior petrosal vein

In the decompressive craniectomy group, the reference CTA score confirmed brain death in 8/10 CTAs (6/8 patients), corresponding to a sensitivity of 80 %, whereas the revised four-point venous CTA score confirmed cerebral circulation arrest in all cases, corresponding to a sensitivity of 100 %.

**Fig. 4:**
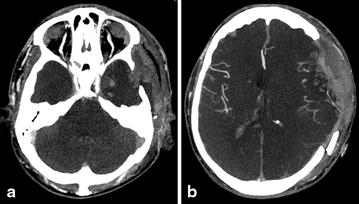
CTA axial views of a clinically brain-dead patient with a craniectomy and no opacification of the SPV (**a**) despite persistent enhancement of the bilateral C-MCA (**b**)

Among the seven patients for whom a second CTA was needed because the first did not confirm circulatory arrest according to the reference CTA score, three had infratentorial haemorrhage, and two had decompressive craniectomy (Fig. 4). The revised four-point venous CTA score analysed with the initial CTA showed no opacification of the ICV and SPV (positive four-point venous CTA score) in five of the seven patients.

Non-opacification of the SPV was observed in 95 % of the patients, corresponding to the high sensitivity of this criterion from the infratentorial circulation. The SPV was still opacified in four CTAs (4/83) (Table 2): two in cerebral anoxia, one with a diffuse cerebral haemorrhage, and one with an ischaemic stroke. The SPV was not opacified in any cases for which the CTA reference score confirmed cerebral circulation arrest. In one case, SPV was still opacified with a lack of ICV opacification (case of cerebral anoxia).

There was no case where only 1 of the 2 ICV or 1 of the 2 SPV was opacified contrary to C-MCA (Table 2).

## Discussion

Our revised four-point venous CTA score relies on the non-opacification of both the ICV and the SPV and is the first score to consider infratentorial circulation within a model of clinical diagnostic criteria. This score provides a high sensitivity of 95 % to confirm brain death diagnosis, which is higher than the previous reference CTA score. This new score is especially highly efficient in cases of decompressive craniectomy, for which it has a sensitivity of 100 %. In addition, the new score eliminated the need to perform a second CTA 6 h after the first in five out of seven cases and should therefore save time in confirming cerebral circulation arrest after the initial CTA. Moreover, the visibility of the ICV and SPV was consistent in healthy patients.

Brain death is a clinical diagnosis [1] that sometimes must be confirmed by ancillary tests. These tests must ideally prove the whole brain death. Regardless of whether they are optional or mandatory, these confirmatory tests must demonstrate high specificity and very high sensitivity. Moreover, an ancillary test should be as minimally invasive as possible, readily available, and safe [25]. CTA has the advantages of wide availability and rapid performance with low operator dependence and low invasivity. However, a main criticism of CTA is its poor sensitivity in cases of clinical brain death with persistent arterial enhancement [26]; this was particularly applicable to early studies with a lack of reliable and relevant diagnostic criteria. However, recent studies have determined a more sensitive diagnostic score [15], which has been accepted by the scientific community [21]. Dupas et al. were the first to describe a diagnostic score based on a seven-point score [8], which Frampas et al. simplified to establish the current four-point reference score based on the lack of opacification of each ICV and C-MCA [15]. Frampas et al. found that the sensitivity of this score (85.7 %) was better than the earlier seven-point score (62.8 %), although a recent study showed a comparable sensitivity of 73 % for both scores [17]. We found a comparable sensitivity of 88 % for the reference four-point score in the present study.

Our revised four-point CTA score focused on venous circulation and provided a sensitivity of 95 %, which is higher than the reference CTA score. The relevance of the venous circulation to confirm circulatory arrest was previously reported by Frampas et al., who showed that the absence of ICV opacification was more sensitive than the absence of C-MCA opacification [15]. Our study confirmed that the absence of ICV opacification was the most sensitive criterion. To explain this result, we hypothesize that if the systemic arterial pressure is sufficient for arterial and superficial vein enhancement, it does not go through the capillary bed and deep veins. Indeed, Asgeirsson et al. showed that for an organ enclosed in a rigid compartment, a tissue pressure increase led exclusively to an increase in venous side resistance with no arterial resistance change [27]. In cases of brain death, the intracranial pressure and deep venous resistance would be sufficiently high to prevent blood or contrast medium from flowing through the ICV. This hypothesis is also supported by the high sensitivity of the hypoperfusion on computed tomography (CT) perfusion images [17]. It could be very seducing to conclude that no venous enhancement means no perfusion in CT. Nevertheless, no study had ever demonstrated this theory to our knowledge.

A unique feature of our revised four-point CTA score, beyond its focus on venous circulation, is that it considers the infratentorial circulation, unlike previous CTA scores, which only analysed the supratentorial circulation and consequently allowing to a whole brain death diagnosis. However, a clinical diagnosis of brain death involves examination of the brain stem only, which is why Joffe et al. emphasized that a brain-dead patient does not need to have irreversible loss of the entire brain to be dead [28]. Indeed, it is known that several encephalic functions may be retained in brain-dead patients [29].

The SPV, which was shown to be consistently visible in healthy patients despite the presence of hypotrophy in 26 % of cases, was selected as the primary criterion in the new score, in combination with the infratentorial circulation and a clinical brain stem examination.

Finally, a main advantage of our revised four-point venous CTA score is its performance in confirming brain death in patients with previous decompressive craniectomy. These patients were a well-known limitation of previous reference CTA scores because of reduced intracranial pressure leading to frequent arterial persistent opacification [16, 30]. In our study, no cases of ICV or SPV opacification were recorded in patients with decompressive craniectomy. We hypothesized that the intracranial arterial pressure decrease was sufficient to enhance cerebral arterial trunks but not to opacify deep veins. This result is of great interest for such cases because until now, invasive conventional angiography was the only relevant cerebral circulation arrest confirmatory test [15, 19, 31, 32].

Our study had several limitations that should be noted. First, this study was realized with a 64-detector scanner and to our knowledge, no study has ever compared 64-, 32-, and 16- or low detector scanners in brain death diagnosis. Indeed, further investigations are necessary to validate our revised four-point venous CTA score in 32- or low detector scanners. Second, variations in the SPV may have led to difficulties in the analysis. Moreover, most of the radiologists do not usually describe this vessel and do not know where is it exactly located. Nevertheless, this limitation is relative if we consider that interpreting CTAs to confirm brain death requires the expertise of a senior radiologist [7]. Finally, in our study, CTA was not compared to another ancillary test, as EEG or else DSA (the reference examination for circulatory arrest), but DSA is an invasive and time-consuming test that is only available in a few reference centres. Moreover, few previous studies have compared CTA to other tests, and we consider in principle the clinical diagnosis of brain death as the gold standard for assessing the performance of an ancillary test [33].

The innovative and highly sensitive venous score we propose may constitute the reference ancillary test for diagnosing brain death. CTA is likely more relevant for brain death management because it allows simultaneous pre-harvesting body scanning, which is very important because it allows critical time savings during organ harvesting [15]. As recommended, a 6-h delay was respected in our study between clinical brain death diagnosis and CTA realization [21]. Nevertheless, it would be interesting to test the sensitivity of our revised four-point venous CTA score when the ancillary test is realized earlier in order to reduce the whole-organ retrieval procedure.

Moreover, in the next few years, technological advances in mobile CT may increase the time savings and avoid haemodynamic instability due to patient transport, thus transforming CTA into a bedside ancillary test [34].

## Conclusion

A revised four-point venous CTA score based on the non-opacification of both the ICV and the SPV is reliable for confirming cerebral circulation arrest in correlation with clinical brain death, with an improved diagnostic performance compared with the previous reference CTA score. Moreover, this revised four-point CTA score offers higher efficiency for patients with decompressive craniectomy.

## Abbreviations

CT: computed tomography
CTA: computed tomography angiography
SPV: superior petrosal vein
ICV: internal cortical vein
C-MCA: middle cerebral artery
DSA: digital subtraction angiography

## References

[1] Wijdicks EF, Varelas PN, Gronseth GS, Greer DM (2010). Evidence-based guideline update: determining brain death in adults: report of the Quality Standards Subcommittee of the American Academy of Neurology. Neurology.

[2] Hwang DY, Gilmore EJ, Greer DM (2013). Assessment of brain death in the neurocritical care unit. Neurosurg Clin N Am.

[3] Nakagawa TA, Ashwal S, Mathur M, Mysore M (2011). Clinical report-guidelines for the determination of brain death in infants and children: an update of the 1987 task force recommendations. Pediatrics.

[4] Link J, Schaefer M, Lang M (1994). Concepts and diagnosis of brain death. Forensic Sci Int.

[5] Sherrington A, Smith M (2012). International perspectives in the diagnosis of brain death in adults. Trends Anaesth Crit Care.

[6] Busl KM, Greer DM (2009). Pitfalls in the diagnosis of brain death. Neurocrit Care.

[7] Welschehold S, Boor S, Reuland K, Thomke F, Kerz T, Reuland A (2012). Technical aids in the diagnosis of brain death: a comparison of SEP, AEP, EEG, TCD and CT angiography. Dtsch Arztebl Int.

[8] Dupas B, Gayet-Delacroix M, Villers D, Antonioli D, Veccherini MF, Soulillou JP (1998). Diagnosis of brain death using two-phase spiral CT. AJNR Am J Neuroradiol.

[9] Monteiro LM, Bollen CW, van Huffelen AC, Ackerstaff RG, Jansen NJ, van Vught AJ (2006). Transcranial Doppler ultrasonography to confirm brain death: a meta-analysis. Intensive Care Med.

[10] Harding JW, Chatterton BE (2003). Outcomes of patients referred for confirmation of brain death by 99mTc-exametazime scintigraphy. Intensive Care Med.

[11] Heiskanen O (1964). Cerebral circulatory arrest caused by acute increase of intracranial pressure. a clinical and roentgenological study of 25 cases. Acta Neurol Scand Suppl.

[12] Jones KM, Barnes PD (1992). MR diagnosis of brain death. AJNR Am J Neuroradiol.

[13] Boulard G, Guiot P, Pottecher T, Tenaillon A (2005). Management of subjects in a state of brain death and the preservation of organs. Ann Fr Anesth Reanim.

[14] Citerio G, Crippa IA, Bronco A, Vargiolu A, Smith M (2014). Variability in brain death determination in Europe: looking for a solution. Neurocrit Care.

[15] Frampas E, Videcoq M, de Kerviler E, Ricolfi F, Kuoch V, Mourey F (2009). CT angiography for brain death diagnosis. AJNR Am J Neuroradiol.

[16] Sawicki M, Bohatyrewicz R, Safranow K, Walecka A, Walecki J, Rowinski O (2014). Computed tomographic angiography criteria in the diagnosis of brain death-comparison of sensitivity and interobserver reliability of different evaluation scales. Neuroradiology.

[17] Shankar JJ, Vandorpe R (2013). CT perfusion for confirmation of brain death. AJNR Am J Neuroradiol.

[18] Taylor T, Dineen RA, Gardiner DC, Buss CH, Howatson A, Pace NL (2014). Computed tomography (CT) angiography for confirmation of the clinical diagnosis of brain death. Cochrane Database Syst Rev.

[19] Braum M, Ducrocq X, Huot JC, Audibert G, Anxionnat R, Picard L (1997). Intravenous angiography in brain death: report of 140 patients. Neuroradiology.

[20] Kotloff RM, Blosser S, Fulda GJ, Malinoski D, Ahya VN, Angel L (2015). Management of the potential organ donor in the ICU: Society of Critical Care Medicine/American College of Chest Physicians/Association of Organ Procurement Organizations Consensus Statement. Crit Care Med.

[21] Leclerc X (2007). CT angiography for the diagnosis of brain death: recommendations of the French Society of Neuroradiology (SFNR). J Neuroradiol.

[22] Matsushima T, Rhoton AL, de Oliveira E, Peace D (1983). Microsurgical anatomy of the veins of the posterior fossa. J Neurosurg.

[23] Tanriover N, Abe H, Rhoton AL, Kawashima M, Sanus GZ, Akar Z (2007). Microsurgical anatomy of the superior petrosal venous complex: new classifications and implications for subtemporal transtentorial and retrosigmoid suprameatal approaches. J Neurosurg.

[24] Rhoton AL (2000). The posterior fossa veins. Neurosurgery.

[25] Young GB, Lee D (2004). A critique of ancillary tests for brain death. Neurocrit Care.

[26] Quesnel C, Fulgencio JP, Adrie C, Marro B, Payen L, Lembert N (2007). Limitations of computed tomographic angiography in the diagnosis of brain death. Intensive Care Med.

[27] Asgeirsson B, Grande PO (1994). Effects of arterial and venous pressure alterations on transcapillary fluid exchange during raised tissue pressure. Intensive Care Med.

[28] Joffe AR (2007). Limitations of brain death in the interpretation of computed tomographic angiography. Intensive Care Med.

[29] Joffe AR, Anton N (2006). Brain death: understanding of the conceptual basis by pediatric intensivists in Canada. Arch Pediatr Adolesc Med.

[30] Escudero D, Otero J, Marques L, Parra D, Gonzalo JA, Albaiceta GM (2009). Diagnosing brain death by CT perfusion and multislice CT angiography. Neurocrit Care.

[31] Vicenzini E, Pro S, Randi F, Pulitano P, Spadetta G, Rocco M (2010). Transcranial Doppler for brain death after decompressive craniectomy: persistence of cerebral blood flow with flat EEG. Intensive Care Med.

[32] Hansen AV, Lavin PJ, Moody EB, Sandler MP (1993). False-negative cerebral radionuclide flow study, in brain death, caused by a ventricular drain. Clin Nucl Med.

[33] Wijdicks EF (2013). Pitfalls and slip-ups in brain death determination. Neurol Res.

[34] Ebinger M, Fiebach JB, Audebert HJ (2015). Mobile computed tomography: prehospital diagnosis and treatment of stroke. Curr Opin Neurol.

